# Editorial: Acquired and Inherited Cardiac Arrhythmias

**DOI:** 10.3389/fphys.2022.947103

**Published:** 2022-06-21

**Authors:** Yael Ben-Haim, Ofer Binah, José Jalife

**Affiliations:** ^1^ Cardiovascular Clinical Academic Group, Molecular and Clinical Sciences Research Institute, St. George’s University of London, St. George’s University Hospitals NHS Foundation Trust, London, United Kingdom; ^2^ Department of Physiology, Biophysics and Systems Biology, Ruth and Bruce Rappaport Faculty of Medicine, The Rappaport Institute, Technion Israel Institute of Technology, Haifa, Israel; ^3^ Centro Nacional de Investigaciones Cardiovasculares (CNIC), Madrid, Spain

**Keywords:** human pluripotent stem cell derived cardiomyocytes, genetic modifier, long QT syndrome, safety pharmacology, computational models, atrial fibrilation, Torsade de pointes, CAVB dog model

Cardiac arrhythmias are associated with a high rate of morbidity and mortality and cause more than 250,000 deaths annually in the United States (Albert and Stevenson, 2016). Recent data from the UK Biobank showed that cardiac rhythm abnormalities are relatively frequent, affecting over 2% of the adult population (Khurshid et al., 2018). These arrhythmias include, in order of decreasing frequency, atrial fibrillation, bradycardia and conduction system disease, as well as supraventricular tachycardias and ventricular tachycardia (Khurshid et al., 2018). Current knowledge of the mechanisms underlying cardiac arrhythmias emerged from extensive basic and translational studies, aiming at improving the diagnosis and treatment of the different conditions. In this Research Topic we included preclinical studies addressing diverse issues in acquired and inherited arrhythmias, anti-arrhythmic drugs, and new therapy approaches; this in order to further define the precise cellular and molecular basis of cardiac arrhythmias and improve the available therapeutic options.

The manuscripts included in this Research Topic report results from studies that use several different experimental models, and all have potential for clinical impact. First, van Weperen et al. report on the electrophysiological effects of severe bradycardia on Torsade de pointes (TdP) (van Weperen et al.). TdP is a potentially life threatening polymorphic ventricular tachycardia occurring on an abnormal QT-prolongation which is frequently associated with bradycardia, especially when in presence of atrio-ventricular block. However, while the phenomenon has been known for almost 50 years (Dessertenne, 1966) until recently, there has been considerable speculation as to the pathophysiology of this arrhythmia. In a systematic and well-conducted study in the dog model with complete atrioventricular block (CAVB), these authors have confirmed the long-held view that severe bradycardia increases the likelihood of arrhythmia development and the severity of arrhythmic events by modifying several electrophysiological parameters, particularly, spatial dispersion of refractoriness in three dimensions, and to a lesser extent, temporal dispersion of repolarization. The clinical relevance of this study is obvious as management of patients with TdP, either drug-induced or otherwise, includes identifying and withdrawing the offending drug(s), replenishing the serum potassium concentration, and infusing intravenous magnesium (1–2 g). In some cases, temporary cardiac pacing may help shorten the QT interval (Yap and Camm, 2003). As discussed by van Weperen et al. increasing the pacing rate reduces arrhythmia generation, as such, some patients with chronic cardiac diseases may benefit by treatment with a pacemaker to prevent ventricular arrhythmogenesis.

Two studies in this Research Topic emphasized the utility of human induced pluripotent stem cell-derived cardiomyocytes (hiPSC-CMs) in current cardiovascular research. Sala et al. investigated the effect of hydroxychloroquine (HCQ) using hiPSC-CMs from healthy donors and long QT syndrome (LQTS) patients (Sala et al.). Their results showed that disease-specific hiPSC-CMs could potentially identify proarrhythmic effects of HCQ. hiPSC-CMs from healthy controls and from asymptomatic carriers of a LQTS genetic variant showed less susceptibility towards HCQ-induced electrical abnormalities than hiPSC-CMs from patients with LQTS phenotype. Their work supports the idea that disease-specific hiPSC-CMs could provide additional information to the data gathered from hiPSC lines from healthy subjects. In turn, this genotype-specific hiPSC-CM response might allow rapid drug safety assessment in a precision medicine approach. However, as recognized by the authors in the Limitations section of their paper, the hiPSC-CMs used in their study were likely to be immature both electrically and structurally. This is important because there is substantial evidence in the literature that the maturation state of hiPSC-CMs determines the response to pharmacologic interventions, and that immature cells have an exaggerated response to the QT prolonging effects of a number of commonly used drugs (Da Rocha et al., 2017). In other words, the maturation state of hiPSC-CMs should be considered in any studies attempting to establish pro-arrhythmia (Da Rocha et al., 2017). An additional important use of hiPSC-CMs is to investigate the effect of common genetic variants on variable expressivity of inherited cardiac conditions, as demonstrated by van den Brink et al. In their original research they studied the effects of the common variant KCNH2-K897T in the potassium channel, human ether-a-go-go-related gene (*hERG*), that has previously been associated with QT interval prolongation. By demonstrating that the common variant studied caused an increased arrhythmogenic response when in *cis* with the rare disease-causing variant they established the consequences of the common variant on disease expressivity. As in Sala and colleagues’ research (Sala et al.), their study advances hiPSC as a suitable model for understanding disease expressivity, with the caveat that cell maturity, or lack thereof, may be an important parameter to consider.

Finally, Sanchez de la Nava et al. demonstrate the potential uses of computational models and artificial intelligence algorithm for analysis of the antiarrhythmic effect of medical therapy of atrial fibrillation (AF) (Sanchez de la Nava et al.). The algorithm included anatomical and electrophysiological data, and was able to identify the combination of channel conductances that promoted arrhythmia initiation and could therefore be used to predict AF inducibility. Hence, this *in silico* model could potentially be used for evaluation of drug effect as well.

In recent years there has been great progress in the understanding of the basis of cardiac arrhythmias as well as development of new research techniques, some highlighted in this Research Topic. We think that implementing these and other techniques would further improve the understanding of the mechanism of acquired and inherited cardiac arrhythmias as well as aid in the development of precision medicine and new treatment options.
